# Comparative analysis of acute and chronic painful temporomandibular disorders: Insights into pain, behavioral, and psychosocial features

**DOI:** 10.1371/journal.pone.0318946

**Published:** 2025-02-25

**Authors:** Adrian Ujin Yap, Jung Hwan Jo, Sunghae Kim, Byeong-min Lee, Ji Woon Park

**Affiliations:** 1 Division of Dentistry, Ng Teng Fong General Hospital and Faculty of Dentistry, National University Health System, Singapore; 2 National Dental Research Institute Singapore, National Dental Centre Singapore and Duke-NUS Medical School, Singapore Health Services, Singapore; 3 Department of Oral Medicine, Seoul National University Dental Hospital, Seoul, Korea; 4 Department of Oral Medicine & Oral Diagnosis, Seoul National University School of Dentistry, Seoul, Korea; 5 Dental Research Institute, Seoul National University, Seoul, Korea; Far Eastern Memorial Hospital, TAIWAN

## Abstract

**Objective:**

The scarcity of literature necessitates further research to differentiate between acute and chronic painful temporomandibular disorders (TMDs). This study compared pain characteristics, oral behaviors, jaw function, and psychosocial distress between TMD patients with acute and chronic pain, examined correlations among variables, and identified factors associated with chronic pain-related TMDs (PT).

**Methods:**

Anonymized data were gathered from consecutive patients seeking TMD treatment at a university-based oral medicine clinic. Axis I diagnoses were made using the Diagnostic Criteria for TMDs, and patients with PT were categorized into acute (AP) and chronic pain (CP) groups. Axis II assessments were performed, evaluating pain characteristics, oral behaviors, jaw functional limitation, somatic symptoms, depression, and anxiety. Statistical analysis utilized chi-square/non-parametric tests and logistic regression (α =  0.05).

**Results:**

Among the 488 PT patients, 34.6% experienced AP and 65.4% had CP. Significant differences were observed in pain intensity, interference, disability, jaw overuse behavior, functional limitation, somatic symptom burden, depression, and anxiety. (CP>  AP). Moderate-to-strong correlations were found in both the AP (*r*_*s*_ =  0.43–0.83) and CP (*r*_*s*_ =  0.46–0.87) groups, although the specific relationships between pain, behavioral, and psychosocial factors differed somewhat. The multivariate regression model revealed that only pain intensity (OR =  1.01) and oral behaviors (OR =  1.06) significantly increased the odds of chronic PT.

**Conclusion:**

Chronic pain was more prevalent in PT patients and associated with greater severity in pain, behavioral, and psychosocial variables. Pain intensity and oral behaviors were linked to an increased likelihood of chronic pain.

## Introduction

Orofacial pain (OFP), defined as discomfort or pain in the face, mouth, or jaw region, is highly prevalent, affecting up to 25% of the general population [1,2]. Moreover, the occurrence of OFP appears to be on the rise, especially among women [3]. According to the International Association for the Study of Pain (IASP), pain can be classified as acute (lasting ≤ 3 months) or chronic (lasting > 3 months) [4]. Acute OFP is mostly odontogenic, while chronic OFP is mainly associated with Temporomandibular Disorders (TMDs) [5,6]. TMDs encompass a range of musculoskeletal conditions involving the temporomandibular joints (TMJs), masticatory muscles, and related structures [6]. In addition to OFP, the other features of TMDs include TMJ sounds, limited jaw movement, and functional impairments [6,7]. Common TMDs can be categorized into pain-related (painful) and intra-articular (dysfunctional) conditions, as outlined by the Diagnostic Criteria for TMDs (DC/TMD) [8]. Painful TMDs, which adhere to the biopsychosocial model of illness, are influenced by various factors, including genetics, gender, age, trauma, oral behaviors, psychological distress, and somatic symptoms [9–11].

Painful TMDs have been linked to increased functional limitations of the jaw, elevated psychological distress, adverse effects on sleep, reduced oral health-related quality of life (OHRQoL), and other physical symptoms [7,12–15]. Despite their significant impacts, research on the differences between acute and chronic TMD pain, as well as the risk factors for chronic painful TMDs, remains scarce [12–16]. The few studies conducted found that psychological factors were more prevalent in chronic TMD pain patients than in those with acute pain. However, these factors did not seem to increase the risk of developing chronic TMD pain, which was instead related to initial pain intensity [16]. Further research with standardized, validated instruments is needed to differentiate between acute and chronic TMD pain and identify factors associated with chronic pain, potentially revealing latent risk factors for the transition from acute to chronic TMD pain.

The dual-axis DC/TMD, along with its stratified reporting framework, enables the standardized assessment and diagnosis of physical TMD conditions (Axis I), as well as the screening of related behavioral and psychosocial factors (Axis II) [8,17,18]. Among the Axis II instruments are the Graded Chronic Pain Scale (GCPS), Oral Behaviors Checklist (OBC), Jaw Functional Limitation Scale (JFLS), Patient Health Questionnaire-15 (PHQ-15), Patient Health Questionnaire-9 (PHQ-9), and General Anxiety Disorder-7 (GAD-7) [18–24].Uniform use of these instruments enhances TMD research by enabling cross-country comparison, data pooling, and evaluation.

With the aforesaid in consideration, the objectives of this study were threefold: (1) to compare the differences in pain characteristics, oral behaviors, jaw function, somatic symptom burden, and psychological distress between patients with acute and chronic TMD pain; (2) to examine the relationships among these variables within individuals experiencing acute and chronic TMD pain; and (3) to determine the behavioral and biopsychosocial factors associated with chronic pain-related TMDs. The research hypotheses were: (a) patients with chronic TMD pain exhibit significantly greater pain-related interference and disability, jaw overuse behavior, jaw functional limitation, somatic symptom burden, depression, and anxiety than those with acute TMD pain; (b) pain, behavioral, and psychosocial variables show more pronounced correlations in individuals with chronic TMD pain; and (c) the likelihood of chronic pain-related TMDs is increased by specific behavioral and biopsychosocial factors.

## Materials and methods

### Study population

This retrospective study was approved by the Institutional Review Board of the hospital (ERI22001), with a waiver for additional informed consent. Patients provided informed written consent to have data from their medical records used in research on their first visit to the hospital. Anonymized data were extracted from a large-scale collaborative study of the phenotyping of East Asian TMD patients from consecutive “first-visit” TMD patients at a university-based oral medicine clinic between January 2019 and 2022. Data collection was part of routine care, with patients voluntarily providing information and consenting to its use in research. Medical records were accessed between January 2022 to January 2023. All patient data was anonymized for privacy during and after data collection. A minimum of 260 patients was required to achieve a 95% confidence level with a 5% margin of error, based on an estimated 65% prevalence of painful TMDs in East-Asian patients and 1000 patients seen during the evaluation period [17].

Inclusion criteria required individuals to be ≥ 19 years old, proficient in Korean, experiencing orofacial pain, and diagnosed with at least one pain-related TMD via DC/TMD. The exclusion criteria included individuals with only non-painful intra-articular TMDs, prior craniofacial trauma or deformities, substance abuse, non-TMD-related pain, significant physical or mental disabilities, and incomplete questionnaires. At the initial appointment, demographic details were collected, and patients completed the Korean DC/TMD Symptom Questionnaire (SQ) along with Axis II assessments, which comprised the GCPS, OBC, JFLS-20, PHQ-15, PHQ-9, and GAD-7 [18–24].

### TMD diagnosis and patient categorization

The essential TMD-related history was obtained using the SQ, which includes fourteen items evaluating five key TMD symptoms over the past 30 days. After answering the SQ and Axis II questionnaires, patients underwent clinical examination by oral medicine specialists trained and calibrated in the DC/TMD protocol.8 The clinical items assessed included pain locations, palpation or movement pain, jaw movements and deviations, and TMJ noises. When significant structural issues, such as tumors, advanced joint degeneration, or disc displacements, were identified, cone-beam computed tomography and/or magnetic resonance imaging were used to confirm intra-articular diagnoses. Patients with pain-related TMDs, specifically those with masticatory muscle myalgia, TMJ arthralgia, and/or headache attributed to TMDs, were classified into acute and chronic pain groups based on the duration of their symptoms.

### Study measures

#### Pain characteristics.

Orofacial pain characteristics, including pain duration, intensity, interference, and disability, were evaluated using the GCPS [19]. It consists of an item on the number of days with pain, three items measuring pain intensity, and four items assessing functional impact over the past 30 days. The number of days with pain was converted into months, and a significant difference in pain duration between the acute and chronic pain groups was confirmed. The intensity of current, worst, and average pain was rated on a 0 (no pain) to 10 (worst pain imaginable) numerical scale, with the mean of these ratings multiplied by 10 to compute the characteristic pain intensity score. Disability days due to orofacial pain interfering with usual activities were recorded. The impact of pain on daily, recreational, social, and work activities was rated on a 0 (no interference) to 10 (unable to carry on any activities) numerical scale, with the mean of these ratings multiplied by 10 to compute the interference score. Total disability points were determined by summing the assigned points for disability days and interference scores. Chronic pain scale grades were subsequently established by integrating the characteristic pain intensity and total disability points, and were classified as follows: Grade 0 (none); Grade I (low-intensity pain without disability); Grade II (high-intensity pain without disability); Grade III (moderately limiting); and Grade IV (severely limiting).

#### Oral behaviors.

Oral behaviors were assessed using the OBC, which comprises twenty-one items related to oral activities over the past 30 days [20]. The items were rated on a 0 (none of the time) to 4 (4 to 7 nights per week or all of the time) frequency scale. Total OBC scores, reflecting the extent of jaw overuse behavior, were computed by summing the scores for all items and classified as follows: normal (0 to 16 points); low (17 to 24 points); and high (25 to 84 points) [25]. Sleeping-state (SA) and waking-state (WA) oral activity scores were calculated by summing the two sleep-related and nineteen wakefulness-related items, respectively.

#### Jaw function.

Jaw functional limitation over the past 30 days was evaluated using the JFLS-20, including a global scale (JFLS-8). It encompasses three subscales, mastication, vertical jaw mobility, and verbal/non-verbal communication [21]. The items were rated on a 0 (no limitation) to 10 (extreme limitation) numerical scale, and global and subscale scores were computed by summing the scores for the specified items. Higher values indicate greater degrees of “jaw function disability” [26].

#### Somatic symptom burden.

The burden of somatic symptoms over the past 30 days was assessed using the PHQ-15, which contains the fifteen most prevalent complaints associated with somatoform disorders [22]. Items include general, musculoskeletal, neurological, cardiovascular, reproductive, gastrointestinal, and other symptoms, rated on a 0 (not bothered at all) to 2 (bothered a lot) severity scale. Total PHQ-15 scores were computed by summing the scores for all items, with cut-points of 5, 10, and 15 indicating low, medium, and high levels of somatic symptom burden, accordingly.

#### Psychological distress.

Depression and anxiety symptoms over the past 2 weeks were evaluated using the PHQ-9 and GAD-7, which involve nine and seven items, correspondingly [23,24]. For both instruments, items were rated on a 0 (not at all) to 3 (nearly every day) frequency scale. Total PHQ-9 and GAD-7 scores were computed by summing the scores for all items. Cut-points of 5, 10, 15, and 20 indicate mild, moderate, moderately severe, and severe depression, while cut-points of 5, 10, and 15 indicate mild, moderate, and severe anxiety.

### Statistical analyses

Statistical analyses were performed using SPSS software version 26.0 (IBM Corporation, Armonk, NY, USA), with a significance level of 0.05. Categorical data were presented as frequencies with percentages and evaluated using the Chi-square test. Continuous data were reported as means with standard deviations (SDs) and as medians with interquartile ranges (IQRs), with normality assessed by the Shapiro-Wilk’s test. As continuous data were not normally distributed, the Mann-Whitney U test and Spearman’s rank correlation were applied. Correlation coefficients (rs) of 0.1, 0.4, 0.7, and 0.9 indicate weak, moderate, strong, and very strong associations between variables, respectively [27]. Univariate and multivariate logistic regression analyses were conducted to determine the factors related to chronic pain-related TMDs. Non-significant variables were isolated and excluded during multivariate modeling through a stepwise procedure, using a significance threshold of p < 0.10. To ensure the model’s validity, both forward and backward selection techniques were employed. The results were expressed as odds ratios (ORs) with corresponding 95% confidence intervals (95% CIs).

## Results

Of the 773 individuals who met the inclusion criteria, 285 were excluded due to incomplete questionnaires or other exclusion criteria. The final sample comprised 488 patients with pain-related TMD, with a mean age of 36.4 years (SD 15.0), and 69.3% were female. Among these, 34.6% experienced acute pain (AP) and 65.4% had chronic pain (CP). Although gender distribution did not differ significantly, the AP group was significantly older (Table 1).

**Table 1:** Demographics, pain characteristics, oral behaviors, and jaw functional limitation scores of the patients with acute and chronic TMD pain.

Variables		All patients	Acute Pain (AP)	Chronic Pain (CP)	P
**Total**	n (%)	488 (100)	169 (34.6)	319 (65.4)	
**Gender** ^a^					0.097
Female	n (%)	338 (69.3)	109 (64.5)	229 (71.8)	
Male	n (%)	150 (30.7)	60 (35.5)	90 (28.2)	
**Age (years)** ^b^	Mean (SD)	36.35 (14.96)	38.52 (15.69)	35.21 (14.45)	**0.023**
	Median (IQR)	32.00 (25.00, 44.00)	34.00 (26.00, 50.00)	31.00 (25.00, 42.00)	
**Pain characteristics**					
*Duration (months)* ^b^	Mean (SD)	36.36 (64.39)	0.83 (0.71)	55.19 (72.95)	** < 0.001**
	Median (IQR)	9.50 (1.00, 48.00)	1.00 (0.00, 1.00)	36.00 (12.00, 60.00)	
*Intensity (CPI)* ^b^	Mean (SD)	36.34 (25.41)	30.57 (28.38)	38.93 (23.21)	**0.001**
	Median (IQR)	36.67 (16.67, 56.67)	26.67 (0.00, 56.67)	40.00 (20.00, 56.67)	
*Interference score* ^b^	Mean (SD)	23.16 (25.99)	18.84 (25.66)	25.44 (25.91)	** < 0.001**
	Median (IQR)	13.33 (0.00, 43.33)	3.33 (0.00, 30.00)	16.67 (0.00, 46.67)	
*Disability points*	Mean (SD)	1.10 (1.76)	0.92 (1.70)	1.19 (1.79)	**0.027**
	Median (IQR)	0.00 (0.00, 2.00)	0.00 (0.00, 1.00)	0.00 (0.00, 2.00)	
*GCPS* ^a^	Grade 0, n (%)	86 (17.6)	53 (31.4)	33 (10.3)	** < 0.001**
	Grade I, n (%)	202 (41.4)	57 (33.7)	145 (45.5)	
	Grade II, n (%)	105 (21.5)	31 (18.3)	74 (23.2)	
	Grade III, n (%)	53 (10.9)	15 (8.9)	38 (11.9)	
	Grade IV, n (%)	42 (8.6)	13 (7.7)	29 (9.1)	
**Oral behaviors (OBC)** ^b^					
*Total OBC score*	Mean (SD)	15.95 (9.33)	13.09 (6.87)	17.47 (10.08)	** < 0.001**
	Median (IQR)	14.50 (10.00, 21.00)	13.00 (8.00, 17.00)	16.00 (11.00, 22.00)	
*Sleeping state*	Mean (SD)	3.54 (2.51)	2.96 (2.35)	3.85 (2.55)	** < 0.001**
	Median (IQR)	4.00 (1.00, 5.00)	3.00 (1.00, 4.50)	4.00 (2.00, 6.00)	
*Waking state*	Mean (SD)	12.41 (8.01)	10.12 (5.92)	13.62 (8.69)	** < 0.001**
	Median (IQR)	11.00 (7.00, 16.00)	10.00 (6.00, 13.00)	12.00 (8.00, 17.00)	
**Jaw functional limitation (JFLS-20)** ^b^					
*Global score*	Mean (SD)	2.14 (1.80)	1.91 (1.76)	2.26 (1.81)	**0.026**
	Median (IQR)	1.88 (0.63, 3.34)	1.53 (0.47, 3.03)	2.14 (0.76, 3.39)	
*Mastication*	Mean (SD)	2.78 (2.34)	2.58 (2.43)	2.89 (2.28)	0.088
	Median (IQR)	2.50 (0.67, 4.50)	1.67 (0.33, 4.33)	2.67 (0.83, 4.50)	
*Mobility*	Mean (SD)	2.72 (2.25)	2.40 (2.22)	2.89 (2.26)	**0.021**
	Median (IQR)	2.50 (0.75, 4.50)	1.75 (0.25, 4.25)	2.75 (1.00, 4.50)	
*Communication*	Mean (SD)	0.92 (1.63)	0.76 (1.58)	1.00 (1.66)	**0.007**
	Median (IQR)	0.13 (0.00, 1.13)	0.00 (0.00, 0.79)	0.25 (0.00, 1.38)	

CPI =  characteristic pain intensity; GCPS =  graded chronic pain scale; OBC =  oral behavior checklist; JFLS =  jaw functional limitation scale. Results of

^a^Chi-square,

^b^Mann-Whitney U tests. Bold indicates P < 0.05.

Tables 1 and 2 show the mean/median scores for pain, behavioral, and psychosocial variables. Significant differences in pain duration, intensity, interference scores, and disability points were observed (CP>  AP). Moderate-to-severely limiting pain (GCPS Grades III and IV) was experienced by 21.0% of the CP group, compared to 16.6% of the AP group. Substantial differences were also noted in scores for total OBC (jaw overuse behavior), sleeping-state oral activity, waking-state oral activity, and global JFLS, as well as in the mobility and communication subscales between the two patient groups (CP>  AP). Additionally, significant differences in somatic symptom burden, depression, and anxiety were evident (CP>  AP). Medium-to-high somatic symptom burden, depression, and anxiety were present in 19.1%, 16.6%, and 15.3% of the CP group, compared to 15.0%, 12.4%, and 8.3% of the AP group.

**Table 2:** Somatic symptom burden, depression, and anxiety scores of the patients with acute and chronic TMD pain.

Variables		All patients	Acute Pain (AP)	Chronic Pain (CP)	P
**Somatic symptom**					
*PHQ-15 score* ^b^	Mean (SD)	5.35 (4.68)	4.56 (4.77)	5.76 (4.58)	** < 0.001**
	Median (IQR)	4.00 (2.00, 8.00)	3.00 (1.00, 6.00)	5.00 (2.00, 8.00)	
*Severity* ^a^	No/minimal, n (%)	249 (51.0)	101 (59.8)	148 (46.4)	**0.019**
	Low, n (%)	156 (32.0)	46 (27.2)	110 (34.5)	
	Medium, n (%)	59 (12.1)	13 (7.7)	46 (14.4)	
	High, n (%)	24 (4.9)	9 (5.3)	15 (4.7)	
**Depression**					
*PHQ-9 score* ^b^	Mean (SD)	4.74 (5.30)	3.99 (4.96)	5.14 (5.43)	**0.004**
	Median (IQR)	3.00 (1.00, 6.32)	2.00 (0.00, 6.00)	4.00 (1.00, 7.00)	
*Severity* ^a^	No/minimal, n (%)	300 (61.5)	116 (68.6)	184 (57.7)	0.164
	Mild, n (%)	114 (23.4)	32 (18.9)	82 (25.7)	
	Moderate, n (%)	37 (7.6)	9 (5.3)	28 (8.8)	
	Moderately severe, n (%)	22 (4.5)	8 (4.7)	14 (4.4)	
	Severe, n (%)	15 (3.1)	4 (2.4)	11 (3.4)	
**Anxiety**					
*GAD-7 score* ^b^	Mean (SD)	4.05 (5.12)	3.25 (4.28)	4.47 (5.47)	**0.003**
	Median (IQR)	2.00 (0.00, 6.00)	3.00 (1.00, 6.00)	4.00 (1.00, 7.00)	
*Severity* ^a^	No/minimal, n (%)	324 (66.4)	123 (72.8)	201 (63.0)	0.084
	Mild, n (%)	101 (20.7)	32 (18.9)	69 (21.6)	
	Moderate, n (%)	41 (8.4)	8 (4.7)	33 (10.3)	
	Severe, n (%)	22 (4.5)	6 (3.6)	16 (5.0)	

PHQ =  the patient health questionnaire; GAD = generalized anxiety disorder. Results of

^a^Chi-square,

^b^Mann-Whitney U tests. Bold indicates P < 0.05.

Table 3 displays the correlations between the various variables for the patients with acute and chronic pain. For the AP group, moderate correlations were found between the following variables: pain duration–intensity/jaw function, pain intensity–disability/jaw function, pain interference-jaw function, pain disability–jaw function, and somatic symptoms–depression/anxiety (rs =  0.43–0.64). Strong correlations were observed for pain intensity–interference/disability and depression–anxiety (rs =  0.71–0.83). For the CP group, moderate correlations were found between the following variables: pain intensity-interference/disability/jaw function, pain interference-jaw function, pain disability-jaw function, somatic symptoms-depression/anxiety, and depression-anxiety (rs =  0.46–0.64). Strong correlations were noted exclusively between pain interference and disability (rs =  0.87). Table 4 reports the results of univariate and multivariate logistic regression analyses. After adjusting for potential confounders, only pain intensity (OR =  1.01; 95% CI =  1.00-1.02) and oral behaviors (OR =  1.06; 95% CI =  1.03–1.09) were associated with increased odds of chronic pain-related TMDs.

**Table 3:** Correlations between the various variables for patients with acute and chronic TMD pain.

TMD pain	Variables	Duration	Intensity	Interference	Disability	OBC	JFLS	PHQ-15	PHQ-9	GAD-7
**Acute pain**	Duration	–	–	–	–	–	–	–	–	–
	Intensity	**0.516** ***	–	–	–	–	–	–	–	–
	Interference	0.361***	**0.771** ***	–	–	–	–	–	–	–
	Disability	0.222**	**0.619** ***	**0.832** ***	–	–	–	–	–	–
	OBC	0.088	0.170*	0.123	0.157*	–	–	–	–	–
	JFLS	**0.426** ***	**0.641** ***	**0.625** ***	**0.558** ***	0.139	–	–	–	–
	PHQ-15	0.222**	0.125	0.179*	0.192*	0.329***	0.209**	–	–	–
	PHQ-9	0.186*	0.115	0.135	0.218**	0.333***	0.150	**0.626** ***	–	–
	GAD-7	0.215**	0.160*	0.215**	0.253**	0.311***	0.156*	**0.577** ***	**0.706** ***	–
**Chronic pain**	Duration	–	–	–	–	–	–	–	–	–
	Intensity	-0.117*	–	–	–	–	–	–	–	–
	Interference	-0.055	**0.636** ***	–	–	–	–	–	–	–
	Disability	-0.081	**0.573** ***	**0.868** ***	–	–	–	–	–	–
	OBC	0.084	0.146**	0.169**	0.108	–	–	–	–	–
	JFLS	-0.090	**0.527** ***	**0.497** ***	**0.460** ***	0.127*	–	–	–	–
	PHQ-15	0.076	0.212***	0.272***	0.234***	0.331***	0.196***	–	–	–
	PHQ-9	0.103	0.173**	0.270***	0.273***	0.343***	0.104	**0.636** ***	–	–
	GAD-7	0.033	0.186**	0.302***	0.267***	0.350***	0.213***	**0.515** ***	**0.636** ***	–

OBC =  oral behavior checklist; JFLS =  jaw functional limitation scale; PHQ =  the patient health questionnaire; GAD = generalized anxiety disorder. Results of Spearman’s correlation. Bold indicates correlation coefficients > 0.40.

*denotes P < 0.05,

**denotes P < 0.01, and

***denotes P < 0.001.

**Table 4:** Factors associated with chronic pain-related TMDs.

	Univariate analysis			Multivariate analysis		
**Variables**	**OR**	**(95% CI)**	**P**	**OR**	**(95% CI)**	**P**
Gender						
*Female*	1.287	(0.831 - 1.994)	0.258			
*Male*	1.00 (Ref.)					
Age	0.991	(0.977 - 1.005)	0.201			
Pain intensity	1.010	(0.999 - 1.022)	0.066	1.010	(1.003 - 1.018)	**0.008**
Pain interference	1.012	(0.993 - 1.031)	0.212			
Pain disability	0.834	(0.655 - 1.063)	0.142			
Oral behaviors	1.049	(1.020 - 1.080)	**0.001**	1.058	(1.032 - 1.085)	** < 0.001**
Jaw functional limitation	0.979	(0.843 - 1.137)	0.782			
Somatic symptom burden	1.012	(0.955 - 1.072)	0.689			
Depression	0.991	(0.928 - 1.059)	0.792			
Anxiety	1.014	(0.946 - 1.086)	0.693			

*OR =  odds ratio; CI =  confidence interval. Results of logistic regression analysis. Bold indicates P < 0.05.*

## Discussion

This study is one of the few that compared pain characteristics, oral behaviors, jaw function, and psychosocial distress between TMD patients with acute and chronic pain, based on the DC/TMD standard. It also examined correlations among the variables and identified factors associated with chronic pain-related TMDs. The first and second research hypotheses were confirmed, as chronic pain was related to greater severity in pain, behavioral, and psychosocial variables, with moderate-to-strong correlations observed among these variables. As the odds of chronic pain were significantly increased by pain intensity and oral behaviors, the third hypothesis was partially supported. The mean age of the patients and predominance of females were consistent with global patterns observed in TMD patients [28,29]. The higher proportion of chronic pain patients reflects the persistent nature of TMDs, while the older age in the AP group may relate to age-related experiential changes [5,6,30].

### Pain characteristics, oral behaviors, and jaw function

Patients with chronic TMD pain reported significantly greater pain intensity, interference, and disability compared to those with acute TMD pain. The International Association for the Study of Pain revised its definition of pain in 2020 to: “An unpleasant sensory and emotional experience associated with, or resembling that associated with, actual or potential tissue damage” [31]. This updated definition recognizes pain even in the absence of obvious organic causes or visible tissue damage. The greater pain intensity, interference, and disability experienced by the CP group could result from the complex interplay of genetic susceptibility, environmental triggers, epigenetic changes, and other biopsychosocial factors, which amplify the central nervous system’s response to peripheral nociception and sensory stimuli, a phenomenon known as central sensitization [32–34].

Chronic TMD pain patients also indicated significantly greater jaw overuse behavior, increased oral activities during both sleep and wakefulness, and more pronounced jaw functional limitation, particularly in jaw opening, speaking, and expressing emotions. The association between TMD pain and oral behaviors remains uncertain and could be due to either a causal relationship or their high co-occurrence [35]. Existing literature does not support a “direct linear causal relationship” but suggests an intricate connection shaped by various risk factors [35,36]. Recent findings by Vrbanović et al. further suggest that oral behaviors are associated with TMD pain intensity rather than its presence, with anxiety and female sex being significant contributing factors [37]. Additionally, causal attribution beliefs may influence this association, as individuals who believed that oral behaviors placed “very much” strain on the masticatory system reported four times more awake bruxism than those who did not perceive these behaviors as harmful [38]. Similarly, Kliangkaeo et al. and Yap et al. determined that TMD pain intensity was related to jaw functional limitation [39,40]. Furthermore, patients with chronic TMD pain may develop adaptive behaviors and compensatory mechanisms that can exacerbate jaw malfunction [41]. Chronic TMD pain is also associated with psychological distress, which can heighten the perception of functional limitations [12,16,].

### Somatic symptoms, depression, and anxiety

Patients with chronic TMD pain had higher levels of somatic symptom burden, depression, and anxiety than those with acute TMD pain. The relationships between TMDs, somatic symptoms or somatization (the expression of psychological distress through physical symptoms), depression, and anxiety are widely acknowledged [42–44]. East Asian patients may be more inclined to somatization than their Western counterparts, as they are culturally socialized to emphasize physical symptoms rather than psychological symptoms due to the stigma associated with mental illness [7,45]. Central sensitization in chronic pain, potentially arising from stress-mediated neuroplasticity and inflammatory neuromodulation, has been proposed as the underlying mechanism for somatization and somatoform disorders [46,47]. This also clarifies the high prevalence of comorbid chronic pain conditions, which have bidirectional relationships with depression and anxiety, in TMD patients [34,48,49]. The reported prevalence of moderate-to-severe somatization among TMD patients ranged from 28.5% to 76.6%, while moderate-to-severe depression was between 21.4% and 60.1% [42]. In contrast, the prevalence of moderate-to-severe psychosocial impairments for the CP group was notably lower, ranging from 15.3% to 19.1%, and was even lower for the AP group, from 8.3% to 15.0%. The disparities can be accounted for by the use of the Primary Care Evaluation of Mental Disorders (PRIME-MD) instruments, specifically the PHQ-15, PHQ-9, and GAD-7, by the DC/TMD, in place of the Symptom Checklist-90-Revised (SCL-90-R) employed by the Research Diagnostic Criteria for TMDs, which tended to classify patients with more severe symptoms [50].

### Correlation and regression analyses

Aside from the relationships between pain duration and pain intensity/jaw function, the correlations among the variables were similar, though varying in strength, for the AP and CP groups. In patients with acute TMD pain, an increase in pain duration during the first 3 months was associated with greater pain severity and jaw function impairments. This was not evident in patients with chronic pain, indicating that beyond 3 months, the continued presence of pain has minimal impact on pain intensity and jaw functional limitation. Therefore, reducing the period of acute TMD pain is essential to alleviate these adverse effects. The connections between pain intensity, interference/disability, and jaw function were expected, given that TMJ and masticatory muscle pain disrupt jaw functioning and, consequently, life activities. This was also reported in other East Asian TMD patients [40]. Inter-relationships between pain variables and oral behaviors, jaw function, somatic symptoms, as well as psychological distress, although generally significant, were weak and aligned with findings from other studies [51,52]. Somatic symptom burden exhibited a moderate correlation with both depression and anxiety, thereby reinforcing the phenomenon of somatization in individuals with TMDs [42,44]. Neuroscience research indicates that dysregulation in brain regions such as the prefrontal cortex, amygdala, and anterior cingulate cortex, combined with imbalances in neurotransmitters including serotonin, norepinephrine, and dopamine, contributes to somatization, depression, and anxiety. Additionally, cognitive processes involving heightened attention and sensitivity to physical symptoms, along with catastrophizing these symptoms, can exacerbate depression and anxiety [53,54].

In the multivariate analysis, pain intensity and oral behaviors were significantly associated with chronic TMD pain, increasing its odds by 1% and 6%, respectively. While the observed odds may seem modest, this study focused on patients with painful TMDs, who typically exhibit greater psychosocial distress and jaw function impairments compared to those with non-painful or no TMD conditions [7,12]. Findings thus point to potential factors contributing to the transition from acute to chronic painful TMDs. Results from earlier studies also identified pain intensity as a factor related to the transition from acute to chronic TMD pain. Similar to the present study, psychological distress was more prevalent in chronic TMD pain, but it did not appear to increase the risk of transition [16]. Oral behaviors have hitherto not been evaluated and appear to exert a six-fold greater influence than pain intensity. The persistence of repetitive oral activities during both sleep and wakefulness may lead to sustained muscle fatigue, microtrauma, and central sensitization, which can facilitate the development and perpetuation of chronic painful TMDs [32,33,55]. Strategies that support coping and encourage physical movement and activity are effective in reducing pain intensity, while education, psychotherapy, biofeedback, muscle relaxation, posture exercises, botulinum toxin injections, and occlusal appliances are used to manage oral behaviors [56,57]. In addition to the aforementioned factors, various genetic and anatomical vulnerabilities, socio-environmental influences, mental and sleep disorders including Munchausen syndrome and obstructive sleep apnea, as well as iatrogenic issues, such as past misdiagnosis, undertreatment, and overtreatment, have also been implicated in TMD pain chronification [32,33,58].

### Study limitations

While the study has many strengths, including the use of the DC/TMD Axis I and II methodology, a large sample size, and multivariate analysis, it also presents certain limitations. Firstly, the cross-sectional design utilized precludes the inference of causality between the variables examined and the presence of acute or chronic pain. Secondly, the study was centered on Korean TMD patients, which restricts the generalizability of the findings. To enhance the study’s external validity, future research should include TMD patient populations from other countries and clinical settings. Thirdly, although the study addressed many variables, it omitted potential contributors to pain chronicity, such as genetic variants, socio-environmental stressors, and sleep issues [59]. Investigating these could provide a more comprehensive understanding of chronic TMD pain mechanisms.

Fourthly, the DC/TMD Axis II instruments depend on patient self-reporting, which may introduce r recall, social desirability, and other biases [60]. Furthermore, the exclusion of patients who did not complete the Axis II questionnaires may introduce selection bias, potentially leading to a sample that is not fully representative of the broader patient population and skewing the findings with respect to demographics or clinical characteristics. Lastly, while the modest odds ratios for chronic pain indicate a limited effect, the influence of pain intensity and oral behaviors is likely greater when considering patients with both painful and non-painful TMDs.

## Conclusion

Approximately two-thirds of the pain-related TMD patients presented chronic pain. Chronic TMD pain patients exhibited significantly higher levels of pain intensity, interference, disability, oral behaviors, and jaw functional limitation compared to those with acute TMD pain. They also experienced greater somatic symptom burden, depression, and anxiety. The specific relationships between pain characteristics, behavioral, and psychosocial factors varied somewhat between patients with acute and chronic TMD pain. Pain intensity and jaw functional limitation increase with the duration of acute, but not chronic TMD pain. For both pain groups, moderate-to-strong correlations were observed between pain intensity, interference, disability, and jaw functional limitation. The relationships between pain variables and oral behaviors, somatic symptoms, and psychological distress, although generally significant, were weak. Pain intensity and oral behaviors were significantly associated with an increased likelihood of chronic pain in patients with pain-related TMDs. Managing pain severity and modifying oral behaviors in these patients could be crucial for reducing pain chronicity and alleviating related psychosocial issues.
